# Inhibition of translation termination by small molecules targeting ribosomal release factors

**DOI:** 10.1038/s41598-019-51977-1

**Published:** 2019-10-28

**Authors:** Xueliang Ge, Ana Oliveira, Karin Hjort, Terese Bergfors, Hugo Gutiérrez-de-Terán, Dan I. Andersson, Suparna Sanyal, Johan Åqvist

**Affiliations:** 1 Department of Cell and Molecular Biology, Biomedical Center, Uppsala University, SE-75124 Uppsala, Sweden; 2 Department of Medical Biochemistry and Microbiology, Biomedical Center, Uppsala University, SE-75124 Uppsala, Sweden

**Keywords:** Biological techniques, Biochemistry

## Abstract

The bacterial ribosome is an important drug target for antibiotics that can inhibit different stages of protein synthesis. Among the various classes of compounds that impair translation there are, however, no known small-molecule inhibitors that specifically target ribosomal release factors (RFs). The class I RFs are essential for correct termination of translation and they differ considerably between bacteria and eukaryotes, making them potential targets for inhibiting bacterial protein synthesis. We carried out virtual screening of a large compound library against 3D structures of free and ribosome-bound RFs in order to search for small molecules that could potentially inhibit termination by binding to the RFs. Here, we report identification of two such compounds which are found both to bind free RFs in solution and to inhibit peptide release on the ribosome, without affecting peptide bond formation.

## Introduction

Bacterial ribosomes with their auxiliary translation factors are major targets for drug intervention by a wide range of different antibiotics. Such compounds are often derived from natural products and inhibit different phases of protein synthesis, including initiation, peptide chain elongation and mRNA-tRNA translocation, or severely increase the error rate of the translation process^1^. Many of these antibiotics bind directly to the ribosome and thereby interfere with specific stages of protein synthesis. There are also examples, such as kirromycin and fusidic acid, which act by binding to elongation factors (EF-Tu and EF-G, respectively) and thereby blocking their release from the ribosome^2^. Among inhibitors that target the ribosome itself, many of them have been shown to bind either near the peptidyl transferase center (PTC), the mRNA decoding site or in the peptide exit tunnel^1,3^. Both the PTC, where the peptide bond formation reaction takes place, and the decoding site, where cognate aminoacyl-tRNAs are matched with the mRNA codon, are also highly conserved throughout the kingdoms of life. This makes it difficult to find molecules that selectively can block only bacterial protein synthesis. Among inhibitors of the elongation factors EF-Tu and EF-G, the only one used clinically is fusidic acid which is administered topically against skin infections^4^.

**Figure 1:**
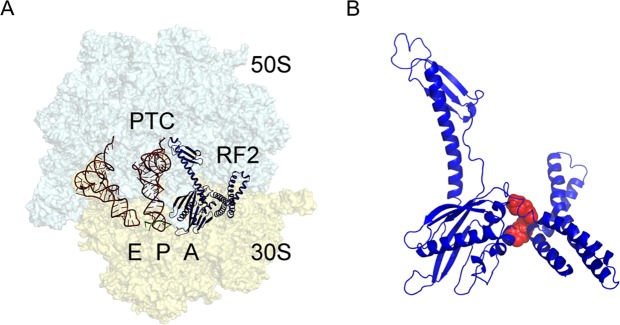
The bacterial 70S ribosome termination complex with RF2. (**A**) View of the ribosome termination complex with E- and P-site tRNAs (brown), mRNA (green) and RF2 (dark blue). (**B**) Close-up view of the hinge region of RF2 between domains 1 and 4 used for virtual screening, where the putative binding region is indicated by a docked ligand (red).

Interestingly, however, no specific small molecule inhibitors of translation termination have ever been reported. Termination of bacterial protein synthesis occurs when a stop codon is presented in the ribosomal A-site and is recognized by a class I release factor, RF1 or RF2. These release factors (RFs) have different but overlapping specificities, where RF1 reads UAA and UAG and RF2 reads UAA and UGA, with strong discrimination against sense codons^5,6^. The RFs are multi-domain proteins, where binding and stop codon recognition by domain 2 at the decoding site causes the universally conserved GGQ motif of domain 3 to insert into the A-site of the PTC, some 80 Å away from the decoding site^7^. This event triggers hydrolysis of the peptidyl-tRNA bond in the P-site of the PTC, and the nascent peptide chain can then be released via the ribosomal exit tunnel (Fig. 1A). In eukaryotes and archaea, on the other hand a single omnipotent RF reads all three stop codons. Although the mechanism of translation termination is basically the same, there is neither sequence nor structural homology between the bacterial RFs and the eukaryotic eRF1, apart from the universally conserved GGQ motif. Hence, it could be expected that putative inhibitors of bacterial RFs and termination, may also selectively inhibit bacteria.

The only known small molecule inhibitor that affects termination is the natural product Blasticidin S (MW = 422), which acts on both prokaryotic and eukaryotic cells and inhibits both peptidyl-tRNA hydrolysis and to a lesser extent peptide bond formation^8^. However, this drug is basically a PTC inhibitor that has been shown to deform the CCA end of the P-site tRNA^8^ and it binds to the ribosomal 50S subunit in a region shared with several other antibiotics^9^. Hence, its mode of action is interference with the P-site tRNA rather than direct interaction with RFs. In contrast, the 18-residue antimicrobial peptide Api137 (i.e., not a small molecule) is a specific inhibitor of termination, with no effect on peptide elongation. It has recently been shown to bind in the peptide exit tunnel and establish a direct interaction with the GGQ motif of RF1^10^. The mode of action here is particularly interesting in that Api137 was shown to enter the exit tunnel after the nascent polypeptide chain has been released, but before the RF has dissociated from the ribosome, thereby trapping the RF on it. The net effect is thus that the pool of free RF1 and RF2 is depleted, causing ribosome stalling at the stop codons^10^. RF1 and RF2 have very high affinity for stop codon programmed ribosomes^11^, with a *K*_d_ ~ 10^−10^ M, and their dissociation is catalyzed by the auxiliary release factor RF3. It is thus conceivable that this high intrinsic affinity could make the class I RFs vulnerable to becoming trapped on the ribosome.

We wanted to explore the possibility that small drug-like molecules might also be able to specifically inhibit translation termination by directly binding to bacterial RFs. To search for such inhibitors, we first carried out computational structure-based screening of a virtual library of commercially available compounds against 3D structures of RF2, which is the major release factor in most bacteria^12^. Hit molecules from this screen were then acquired and tested for their ability to (*i*) bind to free RF2 in solution, (*ii*) inhibit peptide release from the ribosome in an *in vitro* termination assay and (*iii*) arrest bacterial cell growth. Herein, we identify and report two compounds with MW ~ 440 that fulfill these three criteria. These molecules have no effect on peptide chain elongation and are thus the first specific small molecule termination inhibitors, which may open the path for development of a new class of antibiotics.

## Results

### Computational analysis of RF2 as a possible drug target

The release factors RF1 and RF2 acquire an open conformation (Fig. 1A) on the 70S ribosome^13,14^, which is distinctly different from the closed conformation observed in crystal structures of free RFs^15^. The conformational equilibrium of the free RFs in solution, as revealed by SAXS experiments^16^, shows that this open conformation is dominating at about 80%. We explored both conformations of RF2 from *E. coli* and *T. thermophilus* in search of putative binding sites with a series of molecular dynamics (MD) simulations in mixed solvents^17^. By using a water/ethanol solvent mixture of 80/20% mole fraction, this approach allows for identification of preferred hydrophobic and hydrophilic binding regions while also exploring protein flexibility. The MD simulations identified a putative druggable cavity between domains 1 and 4, approximately lined by the first and third α-helix of domain 1 and the C-terminal α-helix of domain 4 (Fig. 1B). This site was selected as the target for virtual screening of 3.4 million drug-like compounds from commercial libraries. These have been filtered with respect to the Lipinski rules, drug-like properties and chemical diversity to yield a structurally diverse selection of lead-like compounds. The virtual screening procedure involves molecular docking of this virtual compound library to a given 3D structure of the target protein. To account for protein flexibility, three representative conformations were explored in parallel by automated docking with the Glide program^18^. These correspond to the open ribosome-bound structure^19,20^, the closed crystal structure of isolated RF2^15,21^ and an intermediate conformation that was found to be stable in the MD simulations. The top 200 ranked compounds were then inspected manually and 60 of them were purchased, based on their chemical diversity and repetitive pattern of interactions within the binding site (Supplementary Table 1).

### Phenotypical screening of bacterial cell growth

To select molecules for further characterization of possible RF binding and termination inhibition, the 60 compounds were initially tested for their minimum inhibitory concentration (MIC) on bacterial growth. This was done using both wild type *Staphylococcus aureus* strain ATCC29213 (Gram^+^) and *Escherichia coli* strain MG1655 (Gram^−^), for which the RF2 protein shows about 45% identity. The initial screening results are given in Table 1 for the eight compounds that were selected for further characterization of their potential interaction with the RFs. All of these (**102, 115**, **118**, **128**, **129**, **137**, **138** and **161**) were found to inhibit growth of *S*. *aureus* and two of them also prevented *E*. *coli* growth (**118** and **129**) at concentrations in the range of 3570 to 3900 mg l^−1^. Each compound was analyzed at different concentrations depending on their solubility in DMSO and their propensity to precipitate in cell culture medium.

**Table 1:** MIC values for the initial set of compounds selected from virtual screening.

Compound	Structure	Properties		MIC	
		MW (g/mol)	Log P	*E. coli* (mg l^−1^)	*S. aureus* (mg l^−1^)
**102**		312.3	1.83	>1560^a^	3120
**115**		441.4	3.41	>500^a^	250
**118**		398.9	1.96	3900	3900
**128**		442.5	1.68	>1050^a^	210
**129**		343.4	1.96	3570	3570
**137**		430.6	3.26	>1070^a^	270
**138**		456.6	2.50	>2280^a^	1140
**161**		442.62	4.48	>500^a^	125

^a^The compound precipitated at higher concentrations in the cell culture media.

### Ligand interaction with RFs in solution

**Figure 2:**
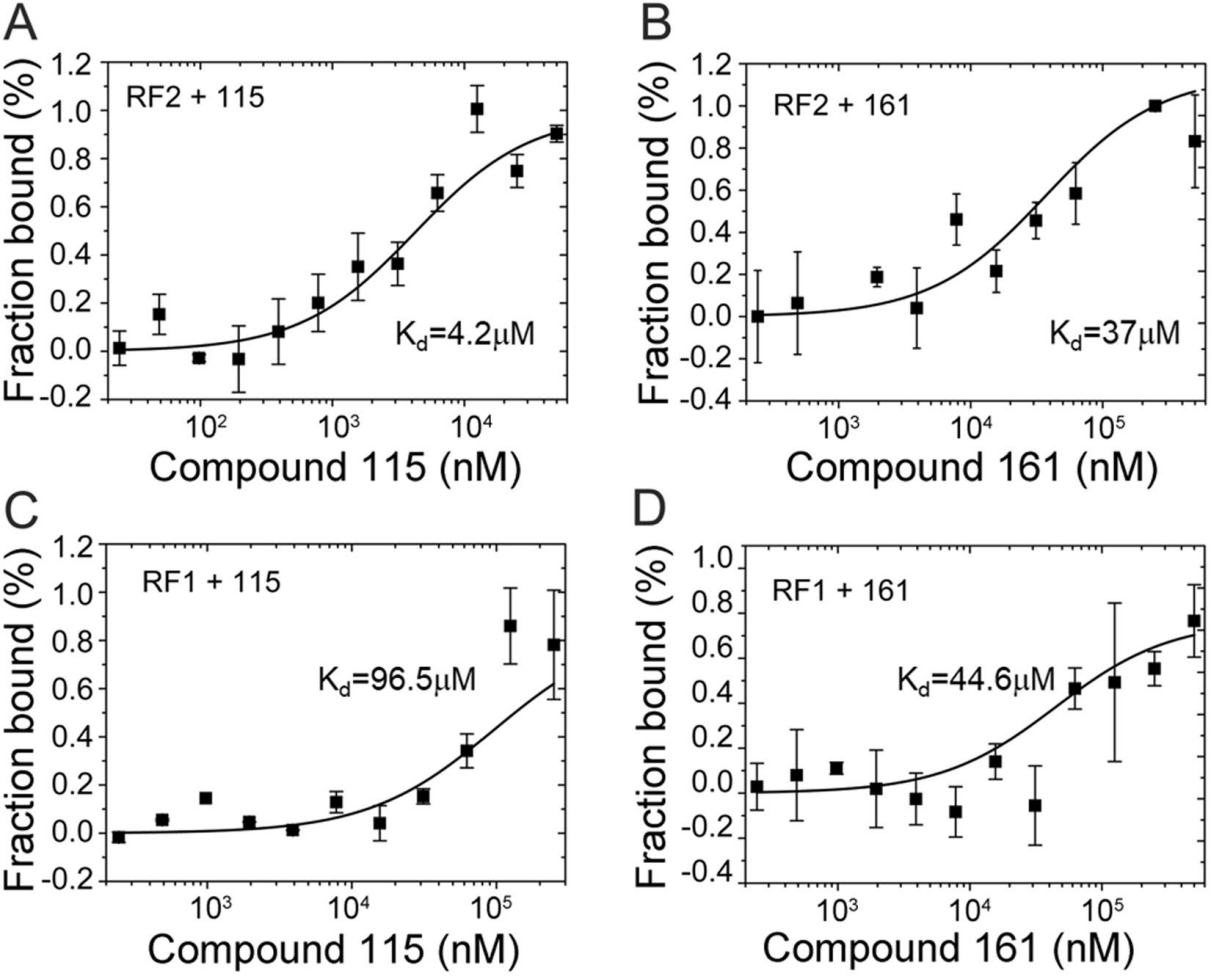
Binding of compounds **115** and **161** to free RF1 and RF2 in solution. Measurement of affinity between *E. coli* release factors – RF2 (**A**,**B**), RF1 (**C**,**D**) and the compounds **115** (**A**,**C**) and **161** (**B**,**D**) by microscale thermophoresis. The resulting binding curves are shown (average of n = 3 ± s.d) from which *K*_d_ values are estimated.

To determine whether the eight selected compounds could bind to RF2 *in vitro*, we tested the compounds in a binding assay with free *E*. *coli* RF2 in solution. Each compound was titrated against 60 nM of the labeled RF2 protein using microscale thermophoresis (MST). Compounds **129**, **137** and **138** did not exhibit binding in the MST assay. Compounds **102**, **118** and **128** on the other hand, showed precipitation at higher concentrations of DMSO than could be tolerated by the RF2 protein (15%) and prevented us to proceed with the experiment. However, two compounds (**115** and **161**) changed the thermophoretic mobility and were found to bind the protein with dissociation constants (*K*_d_) in the μM range. The *K*_d_ determined for ligands **115** and **161** were 4.2 ± 1.7 μM and 37 ± 14 μM, respectively (Fig. 2A,B). These two molecules have a common scaffold, but diverge in terms of their substituents (Table 1), and both can apparently bind to free RF2 in solution. To also compare the affinity of the two best compounds to the homologous RF1 from *E. coli*, we performed the same assay by titrating the compounds against 10 nM of labeled protein. Here, the *K*_d_’s were found to be 96.5 ± 36 μM and 44.6 ± 32 μM, for ligands **115** and **161**, respectively (Fig. 2C,D).

### The inhibitors block turnover of RF2 but do not affect peptide bond formation

**Figure 3:**
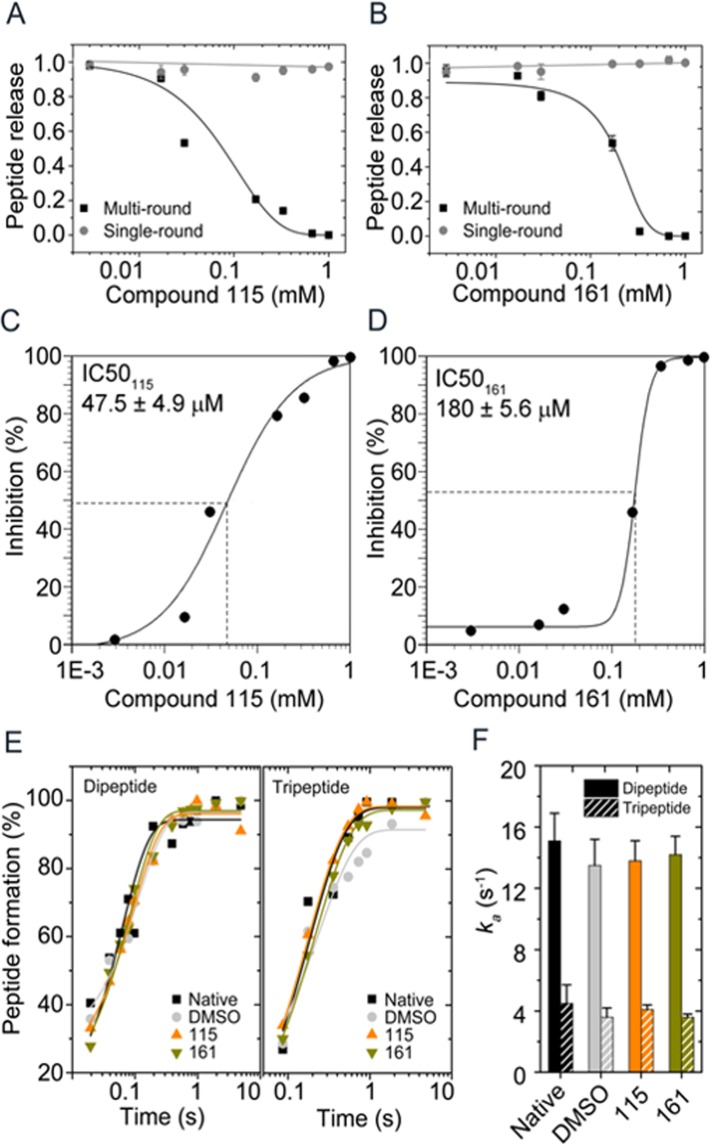
Effects of compounds **115** and **161** on peptide release and di- and tripeptide formation *in vitro*. (**A**,**B**) The effect of the compounds **115** (**A**) and **161** (**B**) on single-round (gray circle) and multiple-round (black square) peptide ([^3^H]fMet) release assays. (**C,D**) Estimation of half maximal inhibitory concentration (IC_50_) for the compounds **115** (**C**) and **161** (**D**) from the results of the multi-round peptide release assay. (**E**) The effect of compounds **115** (orange) and **161** (green) at 0.5 mM and 2% DMSO on fMet-Leu dipeptide and fMet-Leu-Leu tripeptide formation from the quench flow experiment. Apparent rate constants *k*_*a*_ for dipeptide (about 14 s^−1^) and tripeptide (about 4 s^−1^) bond formation are similar in the presence of the **115** and **116** compounds (**F**). All experiments were done in triplicates (average of n = 3 ± s.d).

The fact that the two ligands **115** and **161** evidently can bind bacterial RFs makes it particularly interesting to examine their effect in cell-free translation systems. Based on the MST results, we therefore measured inhibition of translation termination by these two compounds using a fully reconstructed *in vitro* translation system with purified components from *E. coli*. A peptide release complex (RC) was prepared containing mRNA programmed 70S ribosomes carrying [^3^H]fMet-tRNA^fMet^ in the P-site and a UGA stop codon in the A-site. Addition of RF2 to this mix allows release of [^3^H]fMet. The two compounds **115** and **161** were tested with two concentrations of RF2 and with a fixed concentration of RC. In the first single-round peptide release assay, the RF2 concentration was 10-fold higher than that of RC. Under this condition, [^3^H]fMet from all RCs should be released at once and recycling of RF2 will not be needed. No inhibition of single-round peptide release was, however, observed even at a concentration of 1 mM of either of the two compounds (Fig. 3A,B). This indicates that neither **115** nor **161** were able to inhibit the initial association of RF2 with the RC or catalysis of the peptide release reaction.

In the second multiple-round peptide release assay, the concentration of RF2 was 50-fold lower than of that RC, which means that multiple turnover of RF2 is required to release [^3^H]fMet from all RC. Interestingly, in this case, inhibition of peptide release was observed (Fig. 3A,B) and the IC_50_ value for compound **115** was determined to be 47.5 μM (Fig. 3C). From this, the corresponding *K*_i_ was estimated to 3.3 μM, which is very close to the *K*_d_ value determined from MST experiments (*K*_d_ = 4.2 μM). Compound **161** was found to have a higher IC_50_ value of 180 μM (Fig. 3D), which corresponds to a *K*_i_ of 12 μM, again relatively close to the *K*_d_ value of 37 μM obtained from the MST measurements (Fig. 2). These results strongly suggest that both molecules **115** and **161** bind to RF2 on the 70S ribosomal termination complex, thereby inhibiting dissociation and recycling of RF2, which is required for new rounds of translation. Their effect on RF1 catalyzed peptide release was also tested in the multi-round assay, but in that case no significant inhibition was observed (Supplementary Fig. 1).

In order to exclude the possibility that the two compounds might not be specific inhibitors of termination, but instead general inhibitors of the PTC, we tested them also in a di- and tripeptide formation assay^22^. In this experiment, a 70S initiation complex carrying an mRNA coding for Met-Leu-Leu-Stop, and [^3^H]fMet-tRNA^fMet^ in the P-site was rapidly mixed at 37 °C with an elongation mix containing the ternary complex of EF-Tu-Leu-tRNA^Leu^ and EF-G. The compounds **115** and **161** were premixed with the 70S ribosome at varied concentrations. As a control, the solvent DMSO was tested alone. As shown in Fig. 3E,F, no inhibition of dipeptide or tripeptide formation could be seen even at the highest concentration (500 μM) of **115** and **161**. This result confirms that these two compounds are not targeting ribosomes for its peptidyl transferase activity and translocation, but are specific inhibitors of RF2 turnover in the termination process.

### Effect of inhibitors on cell growth

Compounds **115** and **161** were again specifically tested for their effect on bacterial growth using wt *S. aureus*, wt *E*. *coli* and the two *E. coli* mutants *lpxC* and Δ*tolC*, where minimum inhibitory concentrations (MIC) was determined. For *S. aureus*, the MIC for compound **115** was 250 mg l^−1^ (57 μM) and 125 mg l^−1^ (28 μM) for compound **161** (Table 2). For wt *E. coli* the MIC values of compound **115** and **161** were above the highest concentration analyzed, 500 mg l^−1^. Higher concentrations were not possible to test for these specific compounds (**115** and **161**) due to their precipitation in the cell culture media above 500 mg l^−1^. In contrast, the MICs for the *E. coli* drug-hypersensitive *lpxC* mutant and the efflux-defective Δ*tolC* mutant were considerably lower. The *lpxC* mutant showed MICs of 64 mg l^−1^ (14 μM) for compound **115** and 125 mg l^−1^ for compound **161**, while the Δ*tolC* mutant has a MIC of 250 mg l^−1^ for compound **115** and 125 mg l^−1^ for compound **161**. These results suggest that the outer membrane of *E. coli* is a significant barrier for uptake of the compounds, as indicated by the *lpxC* mutant defective in lipid A biosynthesis, and that both compounds are substrates for *TolC*-dependent efflux. As a control, the MIC values for kirromycin were also determined for *S. aureus* and wt *E. coli* and found to be 100 mg l^−1^ and 50 mg l^−1^, respectively.

**Table 2:** MIC values for compounds **115** and **161** in different strains of *E. coli* and *S. aureus* determined by broth micro-dilution.

Strain	MIC (mg l^−1^)	
	115	161
*E. coli* K12 wt	>500^a^	>500^a^
*E. coli ΔtolC*	250	125
*E. coli lpxC*	64	125
*S. aureus* wt	250	125

^a^500 mg l^−1^ was the highest concentration tested due to precipitation in the cell culture media at higher compound concentrations.

**Figure 4:**
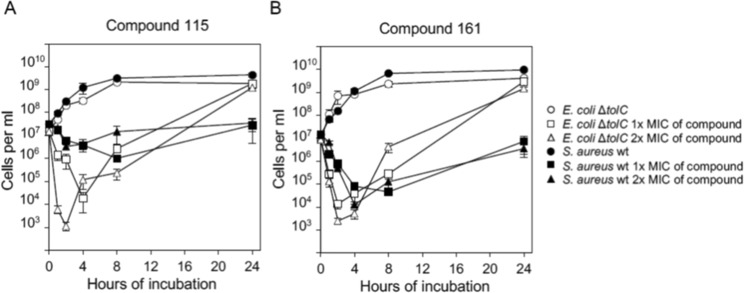
Inhibitors are bactericidal in *S. aureus* and *tolC* defective *E. coli*. Time-kill assays for compound **115** (**A**) and **161** (**B**) for wt *S. aureus* and an *E. coli* Δ*tolC* mutant strain. Cells were incubated with or without compound **115** or **161** for 24 hours. 1x MIC (Minimal Inhibitory Concentration) of compound **115** and **161** are for *E. coli* and *S. aureus* 250 mg l^−1^ and 125 mg l^−1^, respectively. Samples for determination of colony forming units were withdrawn from the time-kill experiment before and 1, 2, 4, 8 and 24 hours after the compound was added. All experiments were done with triplicate biological samples (average of n = 3 ± s.d).

A time-kill assay was further used to determine if the compounds were bacteriostatic or bactericidal. Here, both compounds were found to be bactericidal, that is, the cell number decreased significantly for *S. aureus* and the *tolC*-defective *E. coli* strain during the first four hours of incubation (Fig. 4). However, after about eight hours of incubation, the number of live cells increased in all time-kill assays regardless of bacterial strain or concentration of the inhibitors **115** and **161**. The reason for this regrowth is unclear, but could result from instability of the compounds, emergence of resistant mutants, or regrowth of physiologically adapted cells.

## Discussion

While the ribosomal translation system is the target for many different classes of antibiotics, no small-molecule inhibitors have ever been reported that specifically target the bacterial release factors (RF1 and RF2), which are required for correct and efficient termination of protein synthesis and release of newly synthesized proteins from the ribosome. In principle, the bacterial RFs would appear as interesting drug targets, since their eukaryotic counterpart eRF1 has little sequence homology and a very different 3D structure from RF1 and RF2^6,13–15,23,24^. The only known potent small-molecule inhibitor of bacterial termination is the natural product Blasticidin S produced by *Streptomyces* bacteria, which with an apparent *K*_i_ of about 30 nM, effectively blocks peptide release^8^. However, this compound binds to the PTC of the ribosome^9^ and has been shown to deform the 3’-end of the P-site tRNA such that the tRNA conformation induces steric hindrance for RF binding to the ribosomal A-site^8^. The inhibitory effect of Blasticidin S in termination is thus indirect and does not involve binding to the RFs themselves. Logically, therefore, the molecule also has an inhibitory effect on peptide bond formation (elongation), but with a 6-fold higher *K*_i_ value than for the termination reaction. Moreover, it is not selective for bacteria and it also inhibits translation in mammalian cells^25^, which likely reflects the conserved structure of the P-site on the large ribosomal subunit.

We sought here to identify small drug-like molecules from a large virtual library with binding affinity for the free RFs in solution or bound to the ribosome, with the idea that such compounds could possibly have an effect on translation termination. The strategy was then to combine computational screening (docking) with RF binding experiments and biochemical measurements of the effects of selected compounds on translation termination in a reconstituted cell-free system. All compounds from the virtual screening that were acquired were initially screened for their effect on bacterial growth in *S. aureus* and *E. coli* strains. This was done in order to filter out a smaller subset of potentially interesting molecules for subsequent *in vitro* binding and inhibition experiments. Obviously, this phenotypic screening may yield both false positives and negatives with regard to the ability of the molecules to inhibit termination. However, the same would be true for initial screening of binding to the free RFs in solution since both the target site and the actual target RF conformation are unknown. The strategy employed here was, nevertheless, successful in picking out compounds that both can bind to RF2 and inhibit peptide release. The most potent compounds **115** and **161** have both binding affinities (*K*_d_) to free RF2 and inhibition constants on the ribosome (*K*_i_) in the multiple round peptide release assay in the low μM region. While *K*_i_ values in this range are clearly higher than those typical for tight binding drug molecules, they are unusually good for hits from a first round of virtual screening^26^. Moreover, neither of these molecules have any effect on peptide bond formation or tRNA translocation, which shows that they indeed are specific for termination where they most likely interact directly with the RF.

The fact that neither of the two hits **115** and **161** show any inhibition of peptide release in the single round experiments, but only under multiple round conditions when recycling of RF2 is needed, indicates that they are not able to effectively compete with RF2 binding to the ribosome. This is perhaps not so surprising considering that the affinity of the RFs for the stop codon-programmed ribosome is very high, with *K*_d_ in the sub-nM region^11^, compared to the μM *K*_d_’s measured for the small molecules. Instead it appears that the interaction of the inhibitors with the termination complex is such that RF2 becomes trapped on the ribosome and unavailable for multiple turnovers. This situation is similar to that found for the antibacterial peptide Api137 studied by Wilson, Rodnina and coworkers^10,27^, where binding in the exit tunnel after peptide release traps the RF on the ribosome via a direct interaction with it. It is thus likely that our inhibitors bind to and stabilize this state on the ribosome and thereby prevent conformational changes of the RFs required for their dissociation after peptide release.

It should be emphasized that the present study is not aimed at the development of new antibiotics, but rather at the (*in vitro*) exploration of the bacterial RFs as possible drug targets for such efforts. In particular, we were interested in exploring whether small synthetic molecules would be able to block translation termination through a mechanism involving direct binding to the RFs, which do not appear to have received much attention in antibiotics development so far. Since the results obtained from our combined computational and biochemical approach now show that small drug-like molecules can indeed inhibit termination via such a mechanism, this can be regarded as a proof-of-principle. The fact that the MIC values in the bacterial growth assays are high, as well as the actual mode of action in growth inhibition, is thus not relevant for our present purposes since these experiments were primarily used as a screening tool. A first step in further inhibitor development would rather be to try to improve *in vitro* affinities and inhibition by medicinal chemistry in order to arrive at intrinsically more potent compounds that could eventually be tested for effects on bacterial growth. That is, while the inhibitors discovered here are only moderately potent by normal drug standards, they could serve as useful lead compounds for exploring chemical modifications around this scaffold by organic synthesis.

## Methods

### Protein target site search and virtual screening

Crystal structures of free RF2 in the closed conformation with PDB codes 1GQE^15^ and 2IHR^21^, and structures of the open conformation in complex with the ribosome with PDB codes 5DFE^20^ and 4V9N^19^, were used as input for MD exploration in mixed solvents with the MDmix software^17,28^. Each structure was immersed in a pre-equilibrated solvent box of 20% ethanol and 80% water and counter-ions were added to neutralize the system. Molecular dynamics (MD) simulations were performed with the Amber14 program and force field using periodic boundary conditions and the particle mesh Ewald method for long-range electrostatic interactions^29,30^. The structures were slowly heated for 5 ns to the target temperature (300 K) in the NPT ensemble, followed by an additional 5 ns MD equilibration in the NVT ensemble, which was also used in the subsequent production phase. Five replicates of each system were run for 100 ns each, and frames were collected every 10 ps for subsequent analysis with MDmix, which estimates the probability of binding spots based on the calculated density of ethanol molecules. The output from these calculations yields a series of grids with binding free energy values for the ethanol hydroxyl (hydrophilic) and methyl (hydrophobic) probes, as well as the water occupancy.

A collection of 3.4 M compounds was kindly provided by Prof. Xavier Barril (University of Barcelona). This collection was generated from commercial libraries of six preferred vendors (Specs, Enamine, Life Chemicals, Princeton BioMolecular, Key Organics and Asinex) and further filtered to retain non-reactive and drug-like compounds. The library was prepared for docking using Schrödinger’s Ligprep program^31,32^ and a grid of 10 × 10 × 10 Å was built to comprise the binding site determined by the MDmix calculations. The virtual screening workflow (VSW) in the Schrödinger package (Version 15.3) was used for docking with Glide^18^ and final rescoring with the MM-GBSA method^33^. The top 200 compounds were visually inspected to yield a final selection of 60 compounds to be purchased. The tested anti-RF2 chemical compounds from the virtual screen were purchased from Enamine Ltd., Life Chemicals Inc. and Asinex Corporation (Supplementary Table 1). Ligands were dissolved in dimethyl sulfoxide (DMSO) to an initial working concentration of 5–200 mM depending on the compound.

### Binding assays

A thermal shift assay using the Bio-Rad CFX Connect Real-Time PCR system was used to characterize the proteins (RF1 and RF2) and determine their stability and solubility using the JBS buffer screening kit. With this approach we determined the tolerance of the proteins against 1%, 5%, 8%, 10%, 15%, 20% and 25% concentrations of DMSO. RF-ligand binding measurements were performed using the MicroScaleThermophoresis (MST) Monolith NT.115 equipment (Nano-Temper Technologies). The binding experiments were performed at 20 °C in 0.1 M HEPES buffer and 150 mM NaCl at pH 6.5. The RF2 protein was then fluorescence-labeled using the monolith Protein Labeling Kit RED-NHS (amine reactive). Twelve ligand dilutions between 500 nM and 500 μM were prepared in the same buffer as RF2. Each dilution was mixed with equal volume of l0 μl of 60 nM RF2, with a final DMSO concentration under 3%. The fluorescent molecules were excited with a red laser (650–700 nm) in the MST instrument to monitor the spatial distribution of molecules in the capillary. Thermophoresis was measured in each capillary by locally heating the sample with an infrared laser at 40% excitation for 30 s. Since bound and unbound molecules have a different response, the change in depletion in the presence of the inhibitor can be plotted and used to calculate the bound protein fraction. The dissociation constant (K_d_) was then obtained by fitting the results to a binding isotherm, using the Origin software.

### Peptide release assay

The RC was formed in two steps. First *E. coli* 70S ribosome (2 μM), IF1, IF2 and IF3 (all 2 μM), XR7 Met-UGA mRNA (10 μM) with sequence UAAGGAGGUAUUAA**AUGUGA** (Shine-Dalgarno sequence underlined; coding sequence in bold) was incubated at 37 °C for 5 min in HEPES-polymix buffer at pH 7.5^34^ containing 1 mM GTP, 1 mM ATP, 2 mM magnesium acetate and 0.1 U/mL Ribolock solution. Next, 2 μM [^3^H]fMet-tRNA^fMet^ was added to this mix and incubated for 10 min at 37 °C. An additional 4 mM magnesium acetate was added to stabilize the complex. The complex was then applied to a 1.1 M sucrose cushion (prepared in HEPES-polymix buffer pH 7.5) and centrifuged at 55000 rpm for 3 hours at 4 °C in a S55s rotor in SORVALL M150GX ultracentrifuge. The RC pellets were washed and dissolved in HEPES-polymix buffer (pH 7.5) and stored at −80 °C after shock freezing.

For the peptide release assay, RC and RF2 were pre-incubated for 10 min at 37 °C and then mixed together in the presence or absence of the compounds **115** and **161**. For the multi-round peptide release assay, 125 nM RC was mixed with 5 nM RF2 (active concentration) and the anti-RF2 compounds at 0.003−1 mM, for 5 min at 37 °C. For the single-round peptide release assay, 125 nM RC was mixed with 1.250 μM RF2 (active concentration) and anti-RF2 compounds (0.003–1 mM) for 10 seconds at 37 °C. RF3 was not included in these assays and control experiments were performed in the absence of compounds and in the presence of 2% DMSO, which is the solvent for the anti-RF2 compounds. The reactions were quenched by an adding equal volume of 50% formic acid. The amount of [^3^H]fMet released from the RC was determined by scintillation counting of the supernatant after centrifugation for 15 minutes at 14000 rpm at 4 °C.

### Tripeptide formation assay monitoring peptide bond formation and translocation

Ribosomal activity for peptide bond formation and translocation was tested by di- and tripeptide formation assays^22^. For that, an initiation complex and an elongation mix were first prepared in HEPES-Polymix buffer (pH 7.5). The initiation complex contained *E. coli* 70S ribosome (1 μM), IF1, IF2 and IF3 (all 2 μM), XR7 mRNA encoding Met-Leu-Leu (10 μM) with sequence UAAGGAGGUAUUAA**AUG CUGCUGUAA** and 1 μM [^3^H]-fMet-tRNA^fMet^. The elongation mix was prepared by mixing 10 μM EF-Tu, 10 μM EF-Ts, 10 μM EF-G, 10 μM tRNA^Leu^, 0.5 mM Leu amino acid and 1 unit of Leu-tRNA synthetase. All reactions contained 1 mM GTP and ATP, 10 mM phosphoenol pyruvate, 50 mg/ml pyruvate kinase and 2 mg/ml myokinase. To test the effects, 0.5 mM of the anti-RF2 compounds **115** and **161** was added to both IC and EC together with DMSO as control. Both the mixes were incubated separately at 37 °C for 15 min. The reaction was started by mixing equal volumes of the IC and EC at 37 °C in a quench flow apparatus and was quenched by mixing 17% formic acid after different time points. The peptides were isolated from the ribosome by KOH treatment and applied to reverse phase HPLC separation with in-line radioactivity detection as described earlier^22^.

### Bacterial strains and minimum inhibitory concentration determinations

The four different strains analyzed were wild type (wt) *Staphylococcus aureus (strain* ATCC 29213), wt *Escherichia coli* (strain MG1655, K12), a drug-hypersensitive *E. coli lpxC* mutant D22 (strain MG1655) and an efflux-defective *E. coli* Δ*tolC* mutant (strain MG1655). These were grown in cation-adjusted Mueller Hinton II media (MH II, Nordic Biolabs), the standard medium for antibiotic susceptibility testing. For overnight cell cultures one colony from a MH II (Nordic Biolabs) plate was inoculated in 1 ml MH II media in a 10 ml tube and the bacteria was incubated at 37 °C shaking at 190 rpm. Cells from the overnight culture was used for MIC determinations and time-kill experiments.

The minimal inhibitory concentrations (MIC) of the drugs were measured in 96-well round-bottom microtiter plates using cation-adjusted Mueller-Hinton II (MH II) media. All measurements were done with at least three biological replicates. Briefly, bacterial colonies from a non-selective agar plate were re-suspended in 0.9% NaCl to 0.5 McFarland ($\cong 1.5\times {10}^{8}$ cfu/ml). The cell suspension was further diluted to $\cong 5\times {10}^{5}$ cfu/ml (50 µl of bacterial suspension to 10 ml of MHII) and then 90 µl of cell suspension ($\cong 5\times {10}^{5}$ cfu/l) was added to each well. For the initial screen of all the compounds different concentrations was used depending on the solvability of the compound in 50% DMSO and their propensity to precipitate in the cell culture media. For all compounds the MIC measurements were done in a two-fold dilution series and for compound 115 and 161 the concentration was from 500 mg l^−1^ to 4 mg l^−1^ by adding 10 µl of compound 115 or 161 dissolved in 50% DMSO to water giving a final concentration of DMSO of 5% at the highest compound concentration (500 mg l^−1^) analyzed. The positive control (without compound) also contained 5% DMSO to determine if the DMSO concentration influenced bacterial growth. Plates were covered and incubated without shaking at 37 °C for 16–20 h. The MIC value was determined visually as the lowest concentration of drug that inhibited growth.

### Time-kill experiments

To determine if the compounds are bacteriostatic or bactericidal, time-kill assays were performed with wt *S. aureus* and a Δ*tolC* efflux-defective *E. coli*. The initial inoculums were diluted from triplicate biological replicates of overnight cell cultures inoculated with one colony in 1 ml MH II medium. The cell inoculum was diluted 1:1000 in 1 ml MH II medium and the cell culture was allowed to recover for 30 minutes before the compounds were added at a final concentration of 1x MIC (250 mg l^−1^ for compound **115** and 125 mg l^−1^ for compound **161** for both bacterial strains) or 2x MIC. As a control of the possible effect of DMSO on cell growth, the positive growth controls (three biological replicates) contained the same DMSO concentration as the cells with compound added (2x MIC). Samples (20 µl) were withdrawn before the compound was added and also after 1, 2, 4, 8 and 24 h after addition. The cells were diluted in 0.9% NaCl before they were plated on non-selective agar plates using glass beads. Colonies were counted after approximately 20 h.

## Supplementary information

Supplementary Information

## Data Availability

The data that support the findings of this study are available from the corresponding author upon reasonable request.
