# Author Correction: Inhibition of translation termination by small molecules targeting ribosomal release factors

**DOI:** 10.1038/s41598-020-62010-1

**Published:** 2020-03-13

**Authors:** Xueliang Ge, Ana Oliveira, Karin Hjort, Terese Bergfors, Hugo Gutiérrez-de-Terán, Dan I. Andersson, Suparna Sanyal, Johan Åqvist

**Affiliations:** 1 Department of Cell and Molecular Biology, Biomedical Center, Uppsala University, SE-75124 Uppsala, Sweden; 2 Department of Medical Biochemistry and Microbiology, Biomedical Center, Uppsala University, SE-75124 Uppsala, Sweden

Correction to: *Scientific Reports* 10.1038/s41598-019-51977-1, published online 28 October 2019

The original version of this Article contained a typographical error in the spelling of the author Hugo Gutiérrez-de-Terán, which was incorrectly given as Hugo Gutierrez de Terán. This has now been corrected in the PDF and HTML versions of the Article, and in the accompanying Supplementary Information file.

