# Supplementary material for: Inhibition of translation termination by small molecules targeting ribosomal release factors
Source: Sci Rep. 2019 Oct 28;9:15424. doi: 10.1038/s41598-019-51977-1 (PMC6817905; doi:10.1038/s41598-019-51977-1)
Supplement: Supplementary file 1 — Supplementary Information [file 41598_2019_51977_MOESM1_ESM.docx]

**Inhibition of translation termination by small molecules targeting ribosomal release factors**

Xueliang Ge^1^, Ana Oliveira^1^, Karin Hjort^2^, Terese Bergfors^1^, Hugo Gutiérrez-de-Terán^1^, Dan I. Andersson^2^, Suparna Sanyal^1^, Johan Åqvist^1*^

^1^Department of Cell and Molecular Biology, Biomedical Center, Uppsala University, SE-75124 Uppsala, Sweden

^2^Department of Medical Biochemistry and Microbiology, Biomedical Center, Uppsala University, SE-75124 Uppsala, Sweden





**Supplementary Figure 1. Effects of compounds 115 and 161 on peptide release by RF1.** A multiple-round peptide ([^3^H]fMet) release assay was performed to examine the effect of compounds **115** (orange) and **161** (green) on RF1 catalyzed peptide release. RC (125 nM) was mixed with RF1 (5 nM active concentration) and the anti RF2 compounds at various concentration (0.025−1 mM) for 5 min at 37°C. The control experiments were performed in the absence of compounds and in the presence of 2% DMSO. The results show that compounds **115** and **161** have no significant inhibitory activity on peptide release catalyzed by RF1.

**Supplementary Table 1. List of acquired compounds, vendors and ID numbers.**

| 3-({imidazo[1,2-a]pyridin-2-yl}methyl)-1-[(3R,5S,7s)-adamantan-1-yl]urea | Enamine | CAT#Z56766284 |
| --- | --- | --- |
| N1,N2-bis[(2S)-1-hydroxy-3-phenylpropan-2-yl]benzene-1,2-dicarboxamide | Enamine | CAT#Z56794515 |
| N-[2-(4-chlorophenyl)ethyl]-2-[(4-phenoxyphenyl)formamido]acetamide | Enamine | CAT#Z32450695 |
| 3,4-dichloro-N-[(2R)-2-(4-methoxyphenyl)-2-(morpholin-4-yl)ethyl]benzamide | Enamine | CAT#Z31976244 |
| (2S)-2-[({5,6-dimethyl-4-oxo-3H,4H-thieno[2,3-d]pyrimidin-2-yl}methyl)sulfanyl]-N-[(1R)-1,2,3,4-tetrahydronaphthalen-1-yl]propanamide | Enamine | CAT#Z16858266 |
| N-(4-methoxyphenyl)-2-(2-{[(3-methoxyphenyl)methyl](methyl)amino}acetamido)benzamide | Enamine | CAT#Z52671758 |
| 2-(2-{methyl[(4-oxo-3,4-dihydroquinazolin-2-yl)methyl]amino}acetamido)-N-[(4-methylphenyl)methyl]benzamide | Enamine | CAT#Z52671257 |
| 2-(3-oxo-3,4-dihydro-2H-1,4-benzoxazin-4-yl)-N-{4-[(piperidin-1-yl)methyl]-1,3-thiazol-2-yl}acetamide | Enamine | CAT#Z28013717 |
| (2S)-2-[(2-chlorophenyl)formamido]-3-methyl-N-{4-[(pyrrolidin-1-yl)methyl]-1,3-thiazol-2-yl}butanamide | Enamine | CAT#Z28065406 |
| (2S)-4-methyl-2-[(2-methylphenyl)formamido]-N-{4-[(piperidin-1-yl)methyl]-1,3-thiazol-2-yl}pentanamide | Enamine | CAT#Z28014044 |
| N-(2,2-diphenylethyl)imidazo[1,2-a]pyridine-2-carboxamide | Enamine | CAT#Z29209475 |
| ethyl 4-(4-bromobenzamido)benzoate | Enamine | CAT#Z30248352 |
| 2-[(4-chlorophenyl)formamido]-N-[(2R)-2-(dimethylamino)-2-(4-methoxyphenyl)ethyl]acetamide | Enamine | CAT#Z31662760 |
| (2S)-2-[(4-chlorophenyl)formamido]-N-{[4-(dimethylamino)phenyl]methyl}pentanediamide | Enamine | CAT#Z225007044 |
| 2-{2-[(3R)-3-(1,3-benzothiazol-2-yl)piperidin-1-yl]acetamido}-4H,5H,6H-cyclopenta[b]thiophene-3-carboxamide | Enamine | CAT#Z46487138 |
| 1-(2,3-dihydro-1,4-benzodioxin-6-yl)-3-[2-(1H-imidazol-1-yl)acetyl]urea | Enamine | CAT#Z203972992 |
| 1-[(4-fluorophenyl)methyl]-3-({imidazo[1,2-a]pyridin-2-yl}methyl)urea | Enamine | CAT#Z230999904 |
| 2-[(2,4-dichlorophenyl)formamido]-N-{4-[(4-methylpiperidin-1-yl)methyl]-1,3-thiazol-2-yl}acetamide | Enamine | CAT#Z230264780 |
| 2-[(4-fluorophenyl)formamido]-N-(4-{[(3R)-3-methylpiperidin-1-yl]methyl}-1,3-thiazol-2-yl)acetamide | Enamine | CAT#Z218347340 |
| 2-[(3,4-dichlorophenyl)formamido]-N-(4-{[(3R)-3-methylpiperidin-1-yl]methyl}-1,3-thiazol-2-yl)acetamide | Enamine | CAT#Z218345986 |
| 2-[(4S)-2,5-dioxo-4-phenyl-4-propylimidazolidin-1-yl]-N-(3-nitrophenyl)acetamide | Enamine | CAT#Z14251576 |
| 2-{methyl[(4-oxo-3,4-dihydroquinazolin-2-yl)methyl]amino}-N-(4-phenyl-1,3-thiazol-2-yl)acetamide | Enamine | CAT#Z142422164 |
| 5-chloro-2-fluoro-N-{4-[(4-methylpiperidin-1-yl)methyl]-1,3-thiazol-2-yl}benzamide | Enamine | CAT#Z109478844 |
| 2-[(4-methylphenyl)formamido]-N-(4-{[(3R)-3-methylpiperidin-1-yl]methyl}-1,3-thiazol-2-yl)acetamide | Enamine | CAT#Z218345936 |
| methyl (2S)-2-[(3S)-3-(carbamoylamino)-3-phenylpropanamido]-3-(1H-indol-3-yl)propanoate | Enamine | CAT#Z295450250 |
| 2-[(4-chlorophenyl)formamido]-N-(4-{[(3R)-3-methylpiperidin-1-yl]methyl}-1,3-thiazol-2-yl)acetamide | Enamine | CAT#Z218345978 |
| 2-[(2-chlorophenyl)formamido]-N-{4-[(4-methylpiperidin-1-yl)methyl]-1,3-thiazol-2-yl}acetamide | Enamine | CAT#Z230264784 |
| (2R)-2-[(2,4-dichlorophenyl)formamido]-N-[(2R)-2-(dimethylamino)-2-(3-methoxyphenyl)ethyl]-3-methylbutanamide | Enamine | CAT#Z28554895 |
| (2S)-2-[(4-chlorophenyl)formamido]-N-{4-[(4-methylpiperidin-1-yl)methyl]-1,3-thiazol-2-yl}propanamide | Enamine | CAT#Z230261356 |
| 2-[(2,4-dichlorophenyl)formamido]-N-{4-[(piperidin-1-yl)methyl]-1,3-thiazol-2-yl}acetamide | Enamine | CAT#Z230244590 |
| 2-[(4-tert-butylphenyl)formamido]-N-(4-{[(3R,5S)-3,5-dimethylpiperidin-1-yl]methyl}-1,3-thiazol-2-yl)acetamide | Enamine | CAT#Z2241097159 |
| 2-[(4-chlorophenyl)formamido]-N-{4-[(4-methylpiperidin-1-yl)methyl]-1,3-thiazol-2-yl}acetamide | Enamine | CAT#Z230264830 |
| 2-[(4-chlorophenyl)formamido]-N-(4-{[(3S,5S)-3,5-dimethylpiperidin-1-yl]methyl}-1,3-thiazol-2-yl)acetamide | Enamine | CAT#Z230292178 |
| 2-[(4-chlorophenyl)formamido]-N-{4-[(piperidin-1-yl)methyl]-1,3-thiazol-2-yl}acetamide | Enamine | CAT#Z230244672 |
| 3-[2-(2-fluorophenoxy)ethyl]-1-[(R)-(4-fluorophenyl)(1-methyl-1H-imidazol-2-yl)methyl]urea | Enamine | CAT#Z356183816 |
| 1-[(R)-phenyl({[3-(trifluoromethyl)phenyl]carbamoyl})methyl]piperidine-4-carboxamide | Enamine | CAT#Z44517156 |
| 2-[(2-chlorophenyl)formamido]-N-[(2R)-2-(4-methoxyphenyl)-2-(pyrrolidin-1-yl)ethyl]acetamide | Enamine | CAT#Z31656634 |
| 2-[(3,4-dichlorophenyl)formamido]-N-{4-[(piperidin-1-yl)methyl]-1,3-thiazol-2-yl}acetamide | Enamine | CAT#Z230244698 |
| 1-[2-(2-fluorophenyl)ethyl]-3-[(S)-(4-methoxyphenyl)(1-methyl-1H-imidazol-2-yl)methyl]urea | Enamine | CAT#Z356188016 |
| (2-acetamido-1,3-thiazol-4-yl)methyl 2-[(4-bromophenyl)formamido]acetate | Enamine | CAT#Z19497874 |
| 4-chloro-N-[(2R)-2-(dimethylamino)-2-(4-methoxyphenyl)ethyl]-2-fluorobenzamide | Enamine | CAT#Z117038094 |
| (2S)-2-[(4-chlorophenyl)formamido]-N-[2-(3,4-dimethoxyphenyl)ethyl]propanamide | Enamine | CAT#Z26705092 |
| 2-[(2-{[(2H-1,3-benzodioxol-5-yl)carbamoyl]amino}-2-oxoethyl)(ethyl)amino]-N-(2-methoxyphenyl)acetamide | Enamine | CAT#Z128737994 |
| (2R)-2-[(5-acetamido-2-methoxyphenyl)amino]-N-[(4-methylphenyl)methyl]propanamide | Enamine | CAT#Z219629460 |
| N-[(2S)-2-(dimethylamino)-2-(3-fluorophenyl)ethyl]-2-(phenylformamido)acetamide | Enamine | CAT#Z69103565 |
| 1-[(R)-(4-chlorophenyl)(1-methyl-1H-imidazol-2-yl)methyl]-3-[2-(thiophen-2-yl)ethyl]urea | Enamine | CAT#Z356187490 |
| 1-({imidazo[1,2-a]pyridin-2-yl}methyl)-3-[2-(thiophen-3-yl)ethyl]urea | Enamine | CAT#Z433944386 |
| N-[(2S)-2-(dimethylamino)-2-(4-ethylphenyl)ethyl]-2-[(4-ethoxyphenyl)formamido]acetamide | Enamine | CAT#Z69106885 |
| 2-[(4S)-4-methyl-4-(4-methylphenyl)-2,5-dioxoimidazolidin-1-yl]-N-(4-{[(3R)-3-methylpiperidin-1-yl]methyl}-1,3-thiazol-2-yl)acetamide | Enamine | CAT# Z218342816 |
| 1-{4-[({pyrazolo[1,5-a]pyrimidin-6-yl}methyl)amino]butyl}piperidine-4-carboxamide | Enamine | CAT#Z1077483114 |
| 1-cyclooctyl-3-({imidazo[1,2-a]pyridin-2-yl}methyl)-1-methylurea | Enamine | CAT#Z975257304 |
| ethyl 4-(2-((6-(2,6-dimethylimidazo[1,2-a]pyridin-3-yl)pyridazin-3-yl)thio)acetamido)benzoate | LifeChemicals | CAT# F5270-0483; CAS: 1049356-21-9 |
| N-(6-((4-((2-(1H-indol-3-yl)ethyl)amino)-4-oxobutyl)thio)pyridazin-3-yl)benzamide | LifeChemicals | CAT#F5248-1744; CAS: 1331310-58-7 |
| N-(4-(2-((benzo[d][1,3]dioxol-5-ylmethyl)amino)-2-oxoethyl)thiazol-2-yl)-4-(pyridin-2-yl)piperazine-1-carboxamide | LifeChemicals | CAT#; F5314-0509  CAS: 1049507-98-3 |
| N-(4-chlorobenzyl)-2-(2-((3-fluorobenzyl)thio)-5-(hydroxymethyl)-1H-imidazol-1-yl)acetamide | LifeChemicals | CAT#F2258-1049; CAS: 921802-87-1 |
| N-(4-chlorobenzyl)-2-(2-((2,5-dimethylbenzyl)thio)-5-(hydroxymethyl)-1H-imidazol-1-yl)acetamide | LifeChemicals | CAT#F2258-1054; CAS: 921573-42-4 |
| N-(4-chlorobenzyl)-2-(5-(hydroxymethyl)-2-((4-methylbenzyl)thio)-1H-imidazol-1-yl)acetamide | LifeChemicals | CAT# F2258-1055; CAS: 921876-79-1 |
| 1"-[2-hydroxy-2-(6-methyl-1H-indol-3-yl)ethyl]-7-methoxy-3,4-dihydrospiro[1-benzopyran-2,4"-piperidine]-4-ol | Asinex | CAT#SYN17217274 |
| 3-(2-methoxyphenyl)-N-{2-[(1-methyl-1H-imidazol-2-yl)sulfanyl]ethyl}-1H-pyrazole-5-carboxamide | Asinex | CAT#SYN20045182 |
| 1-(3-{[1-(4-ethoxyphenyl)-2,5-dioxopyrrolidin-3-yl]amino}propyl)piperidine-4-carboxamide | Asinex | CAT#BAS07330412 |
| 1-{4-[2-hydroxy-3-(naphthalen-1-yloxy)propyl]piperazin-1-yl}-3-(naphthalen-1-yloxy)propan-2-ol | Asinex | CAT# BAS00817260 |
